# Clinical Impact of MGMT Promoter Methylation in IDH‐Mutant Gliomas: Influence of Threshold Selection and Clinical Confounding

**DOI:** 10.1002/acn3.70528

**Published:** 2026-09-08

**Authors:** Haihui Jiang, Linxi Cao, Xiaodong Chen, Siqi Tong, Qinghui Zhu, Jun Yang, Song Lin, Yong Cui

**Affiliations:** ^1^ Department of Neurosurgery, Peking University Third Hospital Peking University Beijing China; ^2^ Department of Neurosurgery, Beijing Tiantan Hospital Capital Medical University Beijing China; ^3^ National Clinical Research Center for Neurological Diseases, Center of Brain Tumor Beijing Institute for Brain Disorders and Beijing Key Laboratory of Brain Tumor Beijing China

**Keywords:** IDH‐mutant glioma, low‐grade glioma, MGMT promoter methylation, prognosis, propensity score matching

## Abstract

**Background:**

The clinical relevance of MGMT promoter methylation in IDH‐mutant gliomas remains controversial in the era of molecular classification. We aimed to systematically evaluate its clinical relevance by integrating quantitative assessment, cutoff exploration, and adjustment for clinical confounding.

**Methods:**

A total of 558 patients with WHO grade 2–3 IDH‐mutant gliomas were retrospectively analyzed. MGMT promoter methylation was quantified by pyrosequencing. Survival outcomes were assessed using Kaplan–Meier analysis and multivariable Cox regression. Multiple cutoff thresholds were examined, and PSM was applied to balance baseline characteristics.

**Results:**

Quantitative methylation stratification demonstrated significantly shorter PFS and OS in unmethylated tumors compared with methylated tumors. However, no clear stepwise association between increasing methylation levels and survival was observed. Across cutoff thresholds (9%–25%), MGMT promoter methylation was consistently associated with improved PFS, whereas its association with OS varied and diminished at higher cutoffs. In the unmatched cohort, MGMT promoter methylation was significantly associated with prolonged PFS (HR = 0.53, 95% CI: 0.33–0.85, *p* = 0.009) and OS (HR = 0.39, 95% CI: 0.21–0.75, *p* = 0.005). Following PSM, the direction and magnitude of these associations remained comparable (PFS: HR = 0.47, 95% CI: 0.23–0.98, *p* = 0.044; OS: HR = 0.35, 95% CI: 0.12–1.05, *p* = 0.06), although statistical significance was less consistent.

**Conclusion:**

MGMT promoter methylation is associated with favorable clinical outcomes in IDH‐mutant gliomas. Its prognostic significance appears to be influenced by treatment context, methodological factors, and cohort characteristics. These findings support a context‐dependent interpretation of MGMT methylation and highlight the need for standardized analytical approaches in future studies.

**Abbreviations**
CI: confidence Interval
CNS: central nervous system
EOR: extent of resection
FFPE: paraffin‐embedded
FLAIR: fluid‐attenuated inversion recovery
GTR: gross total resection
HR: hazard ratio
IDH: isocitrate dehydrogenase
KPS: Karnofsky performance scale
MGMT: O^6^‐methylguanine‐DNA methyltransferase
OS: overall survival
PFS: progression‐free survival
PR: partial resection
PSM: propensity score matching
RTV: residual tumor volume
SD: standard deviation
SpTR: supratotal resection
SR: subtotal resection
TMZ: temozolomide
TR: total resection
WHO: World Health Organization

## 1 Introduction

O^6^‐methylguanine‐DNA methyltransferase (MGMT) promoter methylation is one of the most extensively studied molecular biomarkers in diffuse gliomas [1]. By silencing MGMT expression, promoter methylation reduces the ability of tumor cells to repair alkylating DNA damage, thereby enhancing sensitivity to temozolomide and other alkylating agents [2]. In glioblastoma, MGMT promoter methylation has been consistently associated with improved treatment response and prolonged survival, and it is widely regarded as both a predictive and prognostic biomarker in clinical practice [2, 3].

However, the clinical significance of MGMT promoter methylation in IDH‐mutant gliomas remains less clear. With the introduction of the WHO Classification of Tumors of the Central Nervous System, Fifth Edition (WHO CNS 5), diffuse gliomas are increasingly stratified according to their molecular profiles, and IDH‐mutant astrocytomas are now recognized as a biologically and clinically distinct entity from IDH‐wildtype glioblastoma [4]. Several studies have attempted to evaluate the prognostic and predictive value of MGMT promoter methylation in IDH‐mutant gliomas, yet the results have been inconsistent [5, 6, 7, 8, 9]. While some reports suggest that MGMT methylation is associated with improved survival in these tumors [5, 6, 7], others have failed to demonstrate an independent prognostic effect after adjusting for established clinical and molecular factors [8, 9].

Multiple factors may contribute to this inconsistency. First, methodological variability in MGMT methylation assessment may substantially influence study results [10, 11]. Different analytical techniques, including methylation‐specific PCR, pyrosequencing, and immunohistochemistry, vary in sensitivity, quantitative resolution, and biological interpretability [11]. Moreover, the CpG sites analyzed and the cutoff values used to define methylation positivity differ considerably across studies, which complicates direct comparisons and may affect the interpretation of clinical associations [12].

Second, clinical confounding factors may obscure the true prognostic impact of MGMT promoter methylation. Variables such as patient age, tumor grade, extent of resection, and adjuvant treatment are well‐established determinants of outcome in glioma patients and may correlate with MGMT methylation status. In retrospective cohort studies, imbalance in these factors between comparison groups can lead to biased estimates of prognostic effects if not adequately controlled.

Propensity score matching (PSM) has increasingly been used in observational studies to mitigate such confounding by creating balanced cohorts with comparable baseline characteristics [13]. By approximating certain features of randomized comparisons, PSM can provide a more reliable assessment of the independent clinical significance of potential biomarkers.

In the present study, we sought to revisit the prognostic relevance of MGMT promoter methylation in patients with IDH‐mutant gliomas within a well‐characterized clinical cohort. Specifically, we first evaluated the survival impact of different quantitative methylation cutoff values to explore potential threshold effects. We then applied propensity score matching to minimize baseline imbalances between methylated and unmethylated groups and reassessed the prognostic significance of MGMT promoter methylation using multivariable Cox regression analyses before and after matching. Through this approach, we aimed to clarify whether MGMT promoter methylation independently influences survival outcomes in IDH‐mutant gliomas or whether its apparent prognostic effect may be partly attributable to underlying clinical confounders.

## 2 Materials and Methods

### 2.1 Patient Cohort

This study included 558 consecutive patients who underwent surgical resection for gliomas at the Department of Neurosurgical Oncology IV, Beijing Tiantan Hospital (BTH) between March 2013 and October 2021. The inclusion criteria were as follows: (1) availability of comprehensive molecular profiling data; (2) histopathological diagnosis of IDH‐mutant glioma according to the 2021 WHO CNS 5; (3) WHO Grade 2–3 tumors; and (4) complete follow‐up data.

### 2.2 Histopathologic Review and Molecularly Integrated Diagnosis

Histopathological evaluation of all tumor specimens was performed at the Department of Neuropathology of BTH, by a panel of three senior neuropathologists (G.H.D., Z.F.G., and D.H.L.), each with over 20 years of diagnostic experience. All evaluations were conducted independently and in a blinded manner, with the neuropathologists unaware of patients' clinical, radiological, and outcome data. An integrated diagnosis was subsequently established based on combined assessment of histopathological features and molecular biomarkers. The molecular analyses included evaluation of chromosome 1p/19q codeletion status, as well as alterations in IDH1/2, TERT, EGFR, CDKN2A/B, and chromosomes 7 and 10. All gliomas were classified as either astrocytoma or oligodendroglioma according to the 2021 WHO CNS 5 classification scheme [4]. In cases of diagnostic discrepancy, a final diagnosis was reached by consensus among the reviewing neuropathologists. Chromosome 1p/19q status was assessed by fluorescence in situ hybridization (FISH). Genetic alterations in IDH1/2, TERT, EGFR, CDKN2A/B, as well as the status of chromosomes 7 and 10, were determined by DNA sequencing. Detailed experimental procedures and interpretation criteria have been described in our previous studies [14, 15, 16, 17]. The overall diagnostic workflow is illustrated in Figure S1.

### 2.3 MGMT Methylation Analysis

MGMT promoter methylation was assessed by pyrosequencing according to the manufacturer's instructions. Briefly, genomic DNA was extracted from 10 formalin‐fixed, paraffin‐embedded (FFPE) tumor sections (5–8 μm thickness) using the QIAamp DNA FFPE Tissue Kit (Qiagen), followed by purification. DNA concentration and quality were evaluated using a NanoDrop 2000 spectrophotometer, and samples with concentrations ≥ 30 ng/μl were considered eligible. A minimum of 2 μg of DNA was subjected to bisulfite conversion prior to PCR amplification. Bisulfite‐treated DNA was amplified to target the MGMT promoter region, encompassing eight CpG sites (CpG 74–81) located in exon 1 (genomic sequence on chromosome 10 from 131,265,507 to 131,265,544, CGctttgCGtccCGaCGccCGcaggtcctCGCGgtgCG). Pyrosequencing was performed using the MGMT Methylation Detection Kit (Gene Tech). The analyzed sequence was YGTTTTGYGTTTYGAYGTTYGTAGGTTTTYGYGGTGYGTA. DNA quality and concentration were assessed before bisulfite conversion, and pyrosequencing runs were evaluated using built‐in quality metrics provided by the PyroMark Q96 software. Pyrosequencing results were reviewed according to the manufacturer's quality control criteria. All samples were processed using the same standardized workflow and analytical platform throughout the study to minimize potential batch‐related and technical variability. Pyrosequencing reactions were conducted on a PyroMark Q96 system (Qiagen), and methylation levels were quantified using the corresponding PyroMark Q96 software. Additional methodological details have been described previously [12].

### 2.4 Treatment and Follow‐Up

All patients underwent tumor resection at our institution. The extent of resection (EOR) was assessed based on volumetric fluid‐attenuated inversion recovery (FLAIR) imaging. All FLAIR‐hyperintense signal abnormalities were included in the lesion volume and quantified in cubic centimeters (cm^3^). Postoperative tumor volume was evaluated on 1‐month postoperative volumetric FLAIR images, with manual segmentation performed using iPlan Cranial software (Brainlab). EOR was categorized according to residual tumor volume (RTV) as follows: total resection (TR, RTV = 0 mL), subtotal resection (SR, 0 < RTV ≤ 5 mL), and partial resection (PR, RTV > 5 mL) [18]. Supratotal resection (SpTR) was defined as complete removal of all FLAIR abnormalities with a postoperative cavity exceeding the preoperative tumor volume [19, 20, 21]. For analytical purposes, SpTR and TR were grouped as gross total resection (GTR), while SR and PR were classified as non‐GTR.

Following histopathological diagnosis of diffuse glioma, treatment strategies were determined through multidisciplinary team discussions. Radiotherapy was administered at a total dose of 45–60 Gy in 1.8–2.0 Gy fractions, with or without concomitant temozolomide (TMZ, 75 mg/m^2^). Adjuvant chemotherapy was primarily delivered according to the standard Stupp protocol (TMZ, 150–200 mg/m^2^, administered for 5 days every 28‐day cycle), with a minimum of six cycles recommended for patients with adequate tolerance.

After completion of treatment, patients underwent regular follow‐up with contrast‐enhanced brain MRI to monitor for tumor progression. Follow‐up intervals were determined based on tumor grade, typically every 6 months for high‐grade gliomas and annually for low‐grade gliomas.

Progression‐free survival (PFS) was defined as the interval from initial surgical resection to radiographic or clinical evidence of tumor progression. Overall survival (OS) was calculated from the date of surgery to death from any cause or last follow‐up. The follow‐up duration ranged from 1.0 to 144.0 months, with a median of 49.0 months. A total of 115 patients experienced PFS events and 59 patients experienced OS events.

### 2.5 Propensity Score Matching

Baseline characteristics of the 558 enrolled patients are presented in Table 1, demonstrating substantial clinical heterogeneity across the cohort. Given that key clinical variables, such as age, sex, performance status, pathology, tumor grade, extent of resection, and treatment strategy, are known to significantly influence patient outcomes, propensity score matching (PSM) was applied to reduce potential confounding. Propensity scores were estimated through a logistic regression model, incorporating the following covariates: age, sex, pathology, tumor grade, Karnofsky Performance Status (KPS), extent of resection, chemotherapy, and radiotherapy. Patients with MGMT promoter methylation were matched to those without methylation using nearest‐neighbor propensity score matching with 1:1, 1:2, and 1:3 matching ratios without replacement. Caliper widths were set at 0.01 for 1:1 matching and 0.1 for both 1:2 and 1:3 matching analyses. This approach was used to generate a balanced cohort for subsequent survival analyses.

**TABLE 1:** Comparison of clinical data between methylated and unmethylated gliomas before PSM.

Variable	Methylated (*n* = 497)	Unmethylated (*n* = 61)	*p*
Age (year)	41.5 ± 9.5	39.3 ± 11.8	0.107
Gender (*n*, %)			0.976
Male	276 (55.5%)	34 (55.7%)	
Female	221 (44.5%)	27 (44.3%)	
KPS score	83.8 ± 10.2	81.0 ± 11.1	0.046^*^
Location (*n*, %)			0.009^**^
Left hemisphere	252 (50.7%)	21 (34.4%)	
Right hemisphere	177 (35.6%)	34 (55.7%)	
Bilateral	68 (13.7%)	6 (9.8%)	
Tumor size (*n*, %)			0.357
1 lobe	287 (57.7%)	31 (50.8%)	
2 lobes	109 (21.9%)	18 (29.5%)	
3 lobes	67 (13.5%)	10 (16.4%)	
≥ 4 lobes	34 (6.8%)	2 (3.3%)	
Pathology (*n*, %)			< 0.001^***^
1p/19q co‐deleted and IDH‐mut oligodendrogliomas	273 (54.9%)	13 (21.3%)	
IDH‐mut astrocytoma	224 (45.1%)	48 (78.7%)	
Tumor grade (*n*, %)			0.701
WHO 2	338 (68.0%)	40 (65.6%)	
WHO 3	159 (32.0%)	21 (34.4%)	
Extent of resection (*n*, %)			0.346
Supratotal	43 (8.7%)	3 (4.9%)	
Total	211 (42.5%)	21 (34.4%)	
Subtotal	160 (32.2%)	25 (41.0%)	
Partial	83 (16.7%)	12 (19.7%)	
Chemotherapy (*n*, %)			0.011^*^
Yes	374 (75.2%)	46 (75.4%)	
No	75 (15.1%)	15 (24.6%)	
NA	48 (9.7%)	0 (0.0%)	
Radiotherapy (*n*, %)			0.001^**^
Yes	223 (44.9%)	41 (67.2%)	
No	226 (45.5%)	20 (32.8%)	
NA	48 (9.7%)	0 (0.0%)	

Abbreviations: IDH, isocitrate dehydrogenase; KPS, karnofsky performance score; NA, not available; PSM, propensity score matching; WHO, World Health Organization.

^*^ *p* < 0.05.

^**^ *p* < 0.01.

^***^ *p* < 0.001.

### 2.6 Statistical Analysis

Continuous variables are presented as mean ± standard deviation (SD) and were compared using the independent‐samples *t*‐test. Categorical variables were compared using the chi‐square test or Fisher's exact test, as appropriate. Survival outcomes were estimated through the Kaplan–Meier method, and differences between groups were assessed with the log‐rank test. Multivariable analysis was performed via the Cox proportional hazards regression model to identify independent prognostic factors. All statistical analyses and figure generation were conducted using R software (version 4.5.1; R Foundation for Statistical Computing, Vienna, Austria). Survival analyses relied on the survival package, and forest plots were generated using the forestploter package. A two‐sided *p‐*value < 0.05 was considered statistically significant.

## 3 Results

### 3.1 Patient Characteristics and Cohort Description

A total of 558 patients with IDH‐mutant gliomas were included in this study. According to MGMT promoter methylation status determined by pyrosequencing, 497 patients were classified as methylated and 61 as unmethylated.

Baseline clinical characteristics stratified by MGMT methylation status are summarized in Table 1. Before PSM, there were no significant differences between the two groups in terms of age, sex, tumor size, extent of resection, or WHO tumor grade (all *p* > 0.05). However, several variables differed between groups. Patients with MGMT methylation had slightly higher Karnofsky Performance Status (KPS) scores compared with those without methylation (*p* = 0.046). In addition, tumor location differed significantly between the two groups: tumors with MGMT methylation were more frequently located in the left hemisphere, whereas unmethylated tumors were more commonly found in the right hemisphere (*p* = 0.009). Regarding pathological subtype distribution, the methylated group contained a substantially higher proportion of 1p/19q co‐deleted and IDH‐mutant oligodendrogliomas (54.9% vs. 21.3%), while the unmethylated group was predominantly composed of IDH‐mutant astrocytomas (78.7% vs. 45.1%) (*p* < 0.001). Significant differences were also observed in adjuvant treatment patterns, including chemotherapy (*p* = 0.011) and radiotherapy (*p* = 0.001). These baseline imbalances suggested the presence of potential confounding factors that might influence survival analyses.

To minimize such confounding, propensity score matching was subsequently performed. After PSM, 59 matched pairs of patients with methylated and unmethylated tumors were identified. Following matching, all baseline variables were well balanced between the two groups, with no statistically significant differences observed (all *p* > 0.05) (Table 2).

**TABLE 2:** Comparison of clinical data between methylated and unmethylated gliomas after PSM.

Variable	Methylated (*n* = 59)	Unmethylated (*n* = 59)	*p*
Age (year)	40.8 ± 9.1	39.7 ± 11.6	0.572
Gender (*n*, %)			0.579
Male	31 (52.5%)	34 (57.6%)	
Female	28 (47.5%)	25 (42.4%)	
KPS score	81.7 ± 12.7	81.5 ± 10.5	0.937
Location (*n*, %)			0.796
Left hemisphere	22 (37.3%)	21 (35.6%)	
Right hemisphere	29 (49.2%)	32 (54.2%)	
Bilateral	8 (13.6%)	6 (10.2%)	
Tumor size (*n*, %)			0.960
1 lobe	31 (52.5%)	30 (50.8%)	
2 lobes	16 (27.1%)	17 (28.8%)	
3 lobes	9 (15.3%)	10 (16.9%)	
≥ 4 lobes	3 (5.1%)	2 (3.4%)	
Pathology (*n*, %)			1.0
1p/19q co‐deleted and IDH‐mut oligodendrogliomas	13 (22.0%)	13 (22.0%)	
IDH‐mut astrocytoma	46 (78.0%)	46 (78.0%)	
Tumor grade (*n*, %)			1.0
WHO 2	39 (66.1%)	39 (66.1%)	
WHO 3	20 (33.9%)	20 (33.9%)	
Extent of resection (*n*, %)			0.885
Supratotal	2 (3.4%)	3 (5.1%)	
Total	24 (40.7%)	21 (35.6%)	
Subtotal	20 (33.9%)	23 (39.0%)	
Partial	13 (22.0%)	12 (20.3%)	
Chemotherapy (*n*, %)			0.831
Yes	45 (76.3%)	44 (74.6%)	
No	14 (23.7%)	15 (25.4%)	
NA	0 (0.0%)	0 (0.0%)	
Radiotherapy (*n*, %)			0.845
Yes	40 (67.8%)	39 (66.1%)	
No	19 (32.2%)	20 (33.9%)	
NA	0 (0.0%)	0 (0.0%)	

Abbreviations: IDH, isocitrate dehydrogenase; KPS, karnofsky performance score; NA, not available; PSM, propensity score matching; WHO, World Health Organization.

### 3.2 Distribution of MGMT Promoter Methylation Levels

MGMT promoter methylation levels were quantified by pyrosequencing. The median methylation level across the cohort was 23%. To explore the potential quantitative relationship between methylation level and patient prognosis, patients were stratified into four groups according to methylation percentage: < 9% (unmethylated, 61 patients), 9%–18% (moderately methylated, 143 patients), 18%–36% (highly methylated, 204 patients), and > 36% (supra‐highly methylated, 150 patients).

**FIGURE 1:**
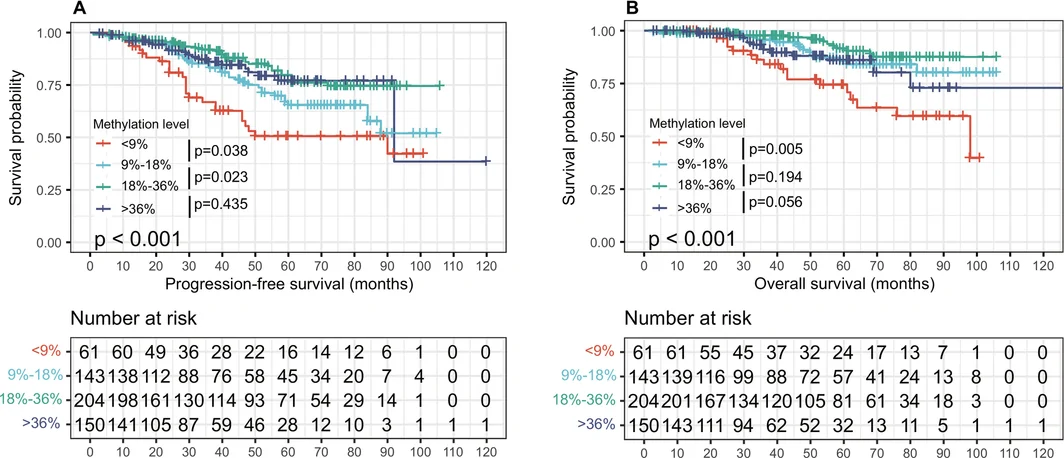
Kaplan–Meier survival curves stratified by the quantitative methylation status. Survival curves for PFS (A) and OS (B) are stratified by quantitative MGMT promoter methylation levels into four groups: < 9%, 9%–18%, 18%–36%, and > 36%. Patients with higher methylation levels demonstrated significantly prolonged PFS and OS compared with those with low methylation (< 9%) (*p* < 0.05). However, no clear stepwise survival gradient was observed across increasing methylation strata.

Kaplan–Meier survival curves demonstrated that patients with unmethylated MGMT promoters had significantly worse PFS and OS compared with those with methylated tumors (Figure 1A,B). However, among tumors with detectable methylation, increasing methylation levels did not demonstrate a clear stepwise association with survival. The 5‐years‐PFS rates were 50.6%, 65.4%, 79.6%, and 77.0%, respectively, while the 5‐years‐OS rates across the four groups were 74.4%, 85.6%, 91.5%, and 86.0%. These findings suggest that the prognostic relevance of MGMT methylation may primarily reflect the presence versus absence of methylation, rather than a quantitative relationship with increasing methylation levels.

### 3.3 Prognostic Implications of Different Methylation Cutoff Values

First, we performed Cox regression analyses treating MGMT methylation percentage as a continuous variable. The results showed that higher methylation levels were significantly associated with improved PFS in the unmatched cohort (HR = 0.984, 95% CI: 0.971–0.997, *p* = 0.014), consistent with the findings from categorical analyses. However, MGMT methylation percentage was not significantly associated with OS in either the unmatched cohort or the post‐PSM cohort (all *p* > 0.05) (Table S1). Therefore, we further performed dichotomized subgroup analyses based on previously reported cutoff values.

**FIGURE 2:**
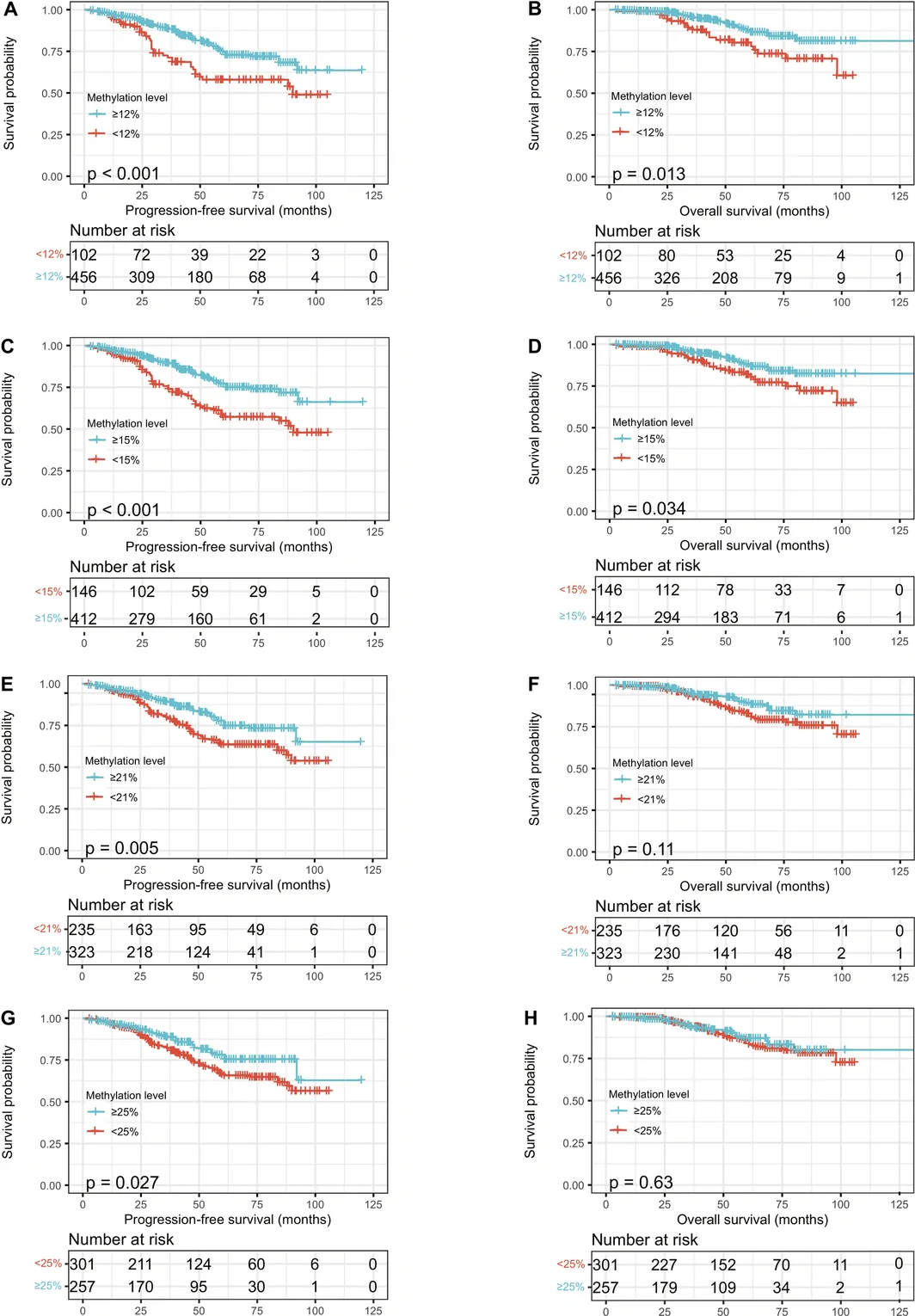
Kaplan–Meier survival curves using various cutoff values. Kaplan–Meier curves for PFS (A, C, E, G) and OS (B, D, F, H) using cutoffs of 12%, 15%, 21%, and 25%. MGMT promoter methylation conferred significantly superior PFS across all tested cutoffs (*p* < 0.05). For OS, the initial benefit seen at lower methylation cutoffs (12%, *p* = 0.013; 15%, *p* = 0.034) progressively attenuated and became nonsignificant at higher cutoffs (21%, *p* = 0.11; 25%, *p* = 0.63).

**FIGURE 3:**
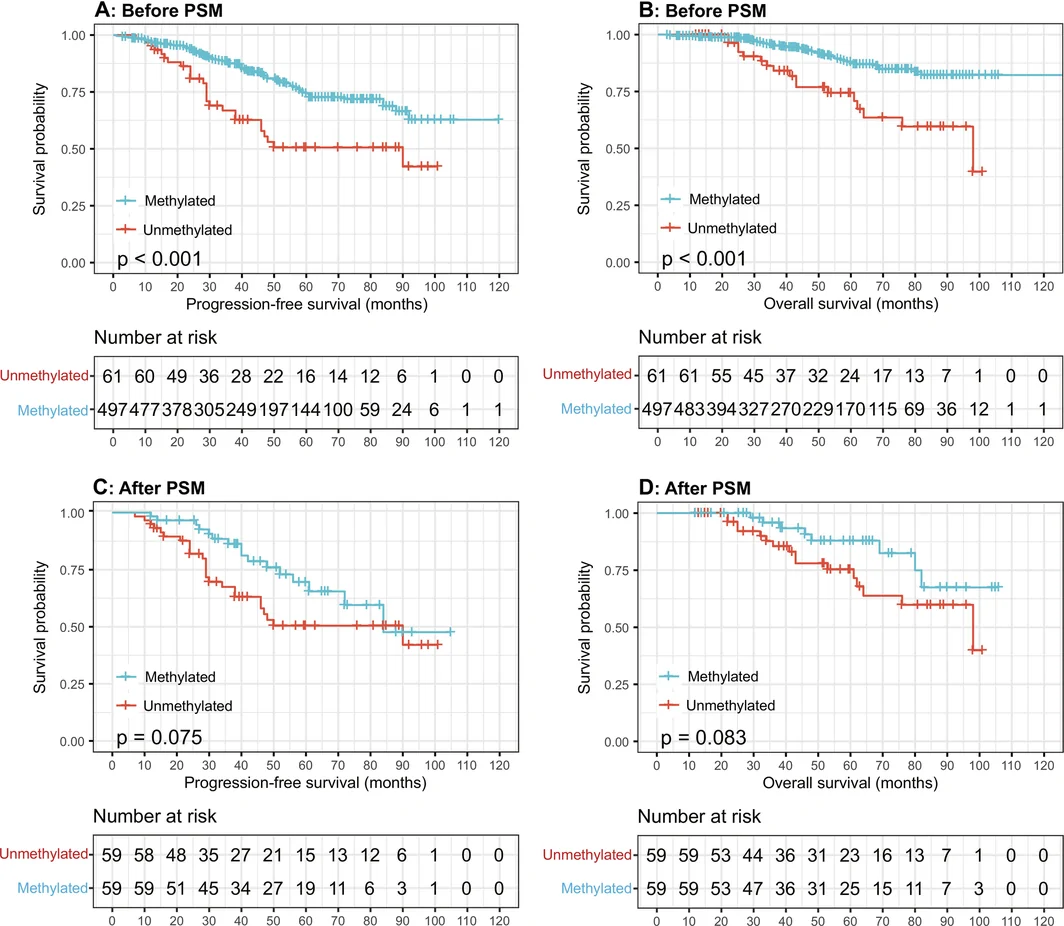
Survival analysis before and after PSM. Kaplan–Meier curves for PFS and OS before (A, B) and after (C, D) PSM. Before matching, MGMT methylation was significantly associated with improved survival (PFS: *p* < 0.001; OS: *p* < 0.001). After matching, patients with MGMT‐methylated tumors continued to demonstrate numerically improved survival; however, the differences did not reach statistical significance (PFS: *p* = 0.075; OS: *p* = 0.083).

Given the heterogeneity of cutoff values reported in previous studies [22, 23, 24, 25, 26], several commonly used thresholds (9%, 12%, 15%, 21%, and 25%) were applied to dichotomize MGMT promoter methylation status. Kaplan–Meier survival analyses comparing methylated and unmethylated tumors under these thresholds are presented in Figure 2 and Figure 3A,B.

Across all tested thresholds, MGMT methylation was consistently associated with improved PFS (all *p* < 0.05). However, the association with OS varied depending on the selected cutoff value. When cutoff values were set at 9%, 12%, and 15%, MGMT methylation was significantly associated with improved OS (9%, *p* < 0.01; 12%, *p* = 0.013; 15%, *p* = 0.034). As the threshold increased to 21% and 25%, the survival difference gradually diminished and became statistically nonsignificant (21%, *p* = 0.11; 25%, *p* = 0.63). These findings indicate that the prognostic value of MGMT promoter methylation may be sensitive to the selected cutoff threshold. Based on prior literature [22] and current clinical practice in our dataset, 9% was selected as the primary cutoff value for subsequent analyses.

### 3.4 Chemotherapy‐Stratified Subgroup Analysis

**FIGURE 4:**
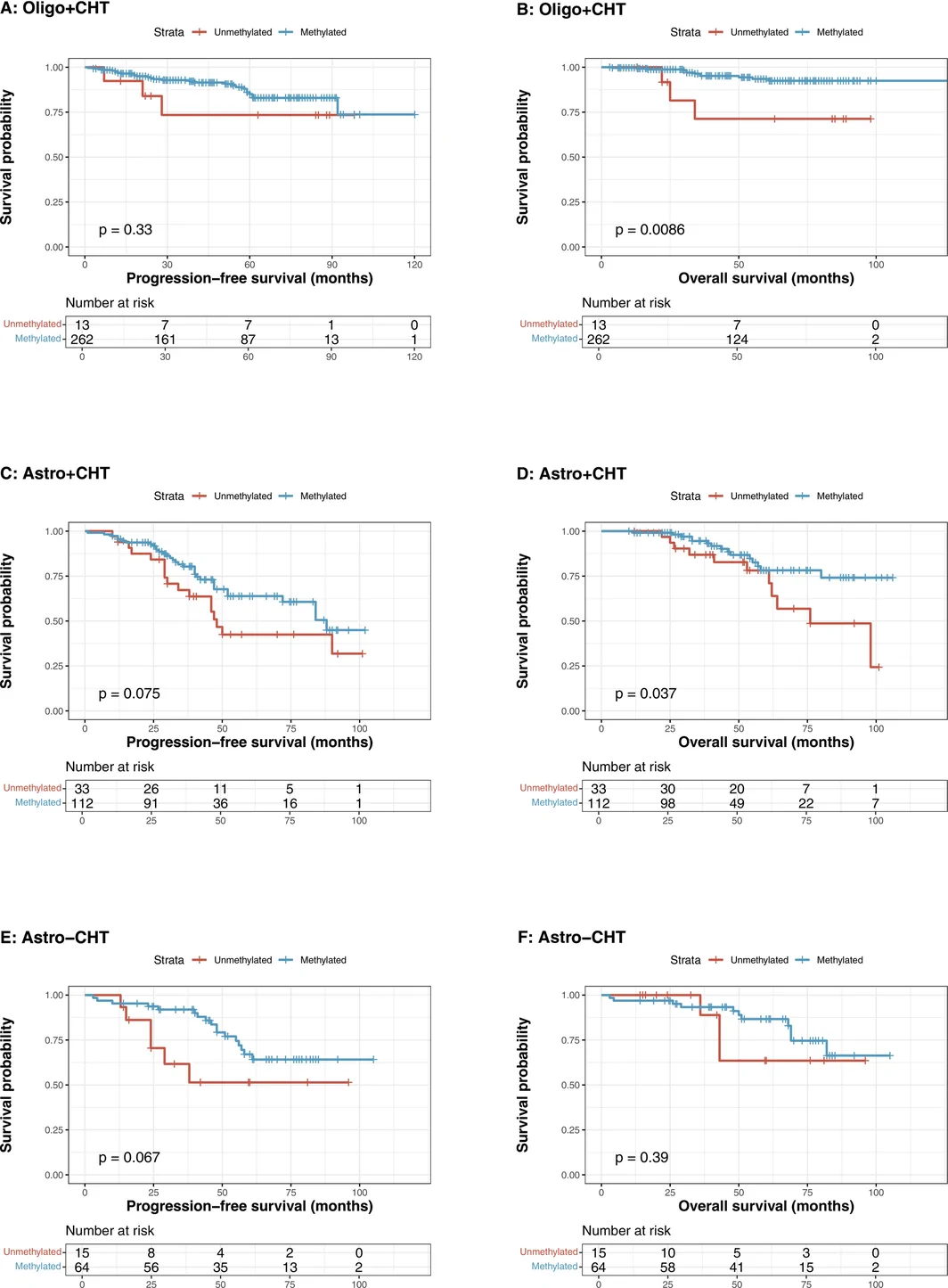
Kaplan–Meier survival curves according to MGMT promoter methylation status across chemotherapy‐stratified subgroups. Kaplan–Meier curves illustrate PFS (left panels) and OS (right panels) according to MGMT promoter methylation status. Survival outcomes were compared between MGMT‐methylated and MGMT‐unmethylated patients in the oligodendroglioma with chemotherapy (Oligo+CHT; A, B), astrocytoma with chemotherapy (Astro+CHT; C, D), and astrocytoma without chemotherapy (Astro−CHT; E, F) cohorts. MGMT promoter methylation was significantly associated with improved OS in the Oligo+CHT (*p* = 0.0086) and Astro+CHT (*p* = 0.037) cohorts, whereas no statistically significant differences were observed in other subgroup comparisons (all *p* > 0.05).

We further performed subgroup analyses stratified by histological subtype and chemotherapy status. Three evaluable cohorts were included: Oligodendroglioma with chemotherapy (Oligo+CHT, *n* = 275), astrocytoma with chemotherapy (Astro+CHT, *n* = 145), and astrocytoma without chemotherapy (Astro−CHT, *n* = 79). The Oligodendroglioma without chemotherapy cohort was excluded due to its limited sample size (*n* = 11), which precluded reliable survival analyses. Kaplan–Meier curves demonstrated that MGMT promoter methylation was associated with significantly improved OS among patients receiving chemotherapy, including those with oligodendroglioma (*p* = 0.0086) and astrocytoma (*p* = 0.037) (Figure 4). In contrast, no significant association between MGMT methylation status and OS was observed in astrocytoma patients who did not receive chemotherapy (*p* = 0.39). For PFS, similar but non‐significant trends toward improved outcomes were observed across all three subgroups, with *p‐*values of 0.33 for Oligo+CHT, 0.075 for Astro+CHT, and 0.067 for Astro−CHT. Subgroup‐specific multivariable Cox regression analyses further supported these findings. MGMT promoter methylation independently predicted favorable OS in the Oligo+CHT cohort (HR = 0.262, 95% CI: 0.076–0.909, *p* = 0.035) and Astro+CHT cohort (HR = 0.447, 95% CI: 0.205–0.974, *p* = 0.043), but was not independently associated with OS in the Astro−CHT cohort (HR = 0.578, 95% CI: 0.160–2.085, *p* = 0.402) (Table 3). Collectively, the Kaplan–Meier and multivariable analyses suggest that the prognostic association of MGMT promoter methylation is mainly observed among patients receiving chemotherapy, further supporting its potential role as a predictive biomarker for chemotherapeutic response.

**TABLE 3:** Multivariable analysis of MGMT in different subgroups.

MGMT	Hazard ratio	95% confidence interval	*p*
In terms of PFS			
Oligo+CHT	0.641	0.197–2.088	0.461
Astro+CHT	0.592	0.329–1.065	0.080
Astro‐CHT	0.426	0.166–1.091	0.075
In terms of OS			
Oligo+CHT	0.262	0.076–0.909	0.035
Astro+CHT	0.447	0.205–0.974	0.043
Astro‐CHT	0.578	0.160–2.085	0.402

Abbreviations: Astro+CHT, astrocytoma with chemotherapy; Astro−CHT, astrocytoma without chemotherapy; Oligo+CHT, oligodendroglioma with chemotherapy; OS, overall survival; PFS, progression‐free survival.

### 3.5 Survival Analysis After Propensity Score Matching

To further address potential confounding caused by baseline heterogeneity, propensity score matching was performed. A total of 59 matched pairs of patients with methylated and unmethylated tumors were identified.

Kaplan–Meier survival curves for the matched cohort are shown in Figure 3C,D. Following propensity score matching, patients with MGMT promoter methylation continued to demonstrate numerically improved survival (PFS: *p* = 0.075; OS: *p* = 0.083) compared with those without methylation. The direction and magnitude of the associations remained comparable to those observed in the unmatched cohort (PFS: HR = 0.570, 95% CI: 0.304–1.071, *p* = 0.081; OS: HR = 0.480, 95% CI: 0.205–1.123, *p* = 0.090), although the associations no longer reached statistical significance after matching.

To further evaluate whether the attenuated statistical significance was primarily attributable to reduced sample size after matching, we performed additional PSM analyses using 1:2 and 1:3 matching ratios. Similar trends were observed, with no statistically significant survival differences detected in either matched cohort. In the 1:2 matched cohort, the *p‐*values were 0.11 for PFS and 0.13 for OS, whereas in the 1:3 matched cohort, the corresponding *p‐*values were 0.19 and 0.13, respectively (Figure S2, Table S2 and Table S3). These findings indicate that, after adjustment for baseline covariates using PSM, MGMT promoter methylation was not independently associated with survival outcomes in the matched cohorts.

### 3.6 Multivariable Cox Regression Analysis

**FIGURE 5:**
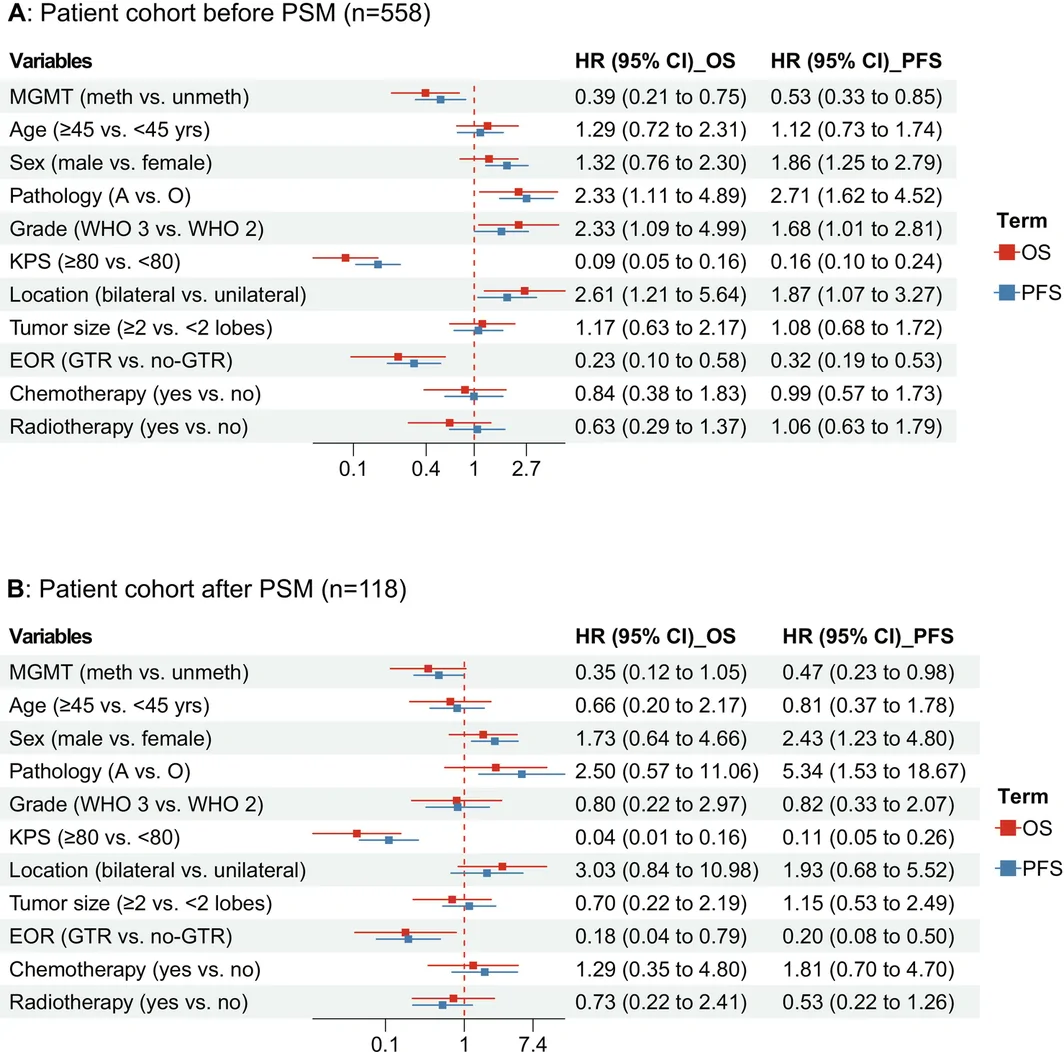
Forest plots from multivariable analysis before and after PSM. Forest plots of multivariable analysis incorporating MGMT promoter methylation, age, sex, pathology, tumor grade, KPS, tumor location, tumor size, extent of resection, and treatment in the unmatched (A) and matched (B) cohorts. MGMT methylation was an independent prognostic factor before PSM (*p* < 0.01). After propensity score matching, the independent prognostic significance of MGMT promoter methylation for OS was marginal (*p* = 0.060), whereas it retains independent significance for PFS (*p* = 0.044).

Multivariable Cox regression analyses were performed to identify independent prognostic factors for survival outcomes (Figure 5). In the unmatched cohort, MGMT promoter methylation, KPS ≥ 80, gross total resection (GTR), WHO Grade 2, and bilateral tumor involvement were independently associated with both OS and PFS (all *p* < 0.05). MGMT promoter methylation was associated with a 61% reduction in the risk of death (OS: HR = 0.39, 95% CI: 0.21–0.75, *p* = 0.005) and a 47% reduction in the risk of tumor progression (PFS: HR = 0.53, 95% CI: 0.33–0.85, *p* = 0.009). After propensity score matching, MGMT promoter methylation remained significantly associated with prolonged PFS (HR = 0.47, 95% CI: 0.23–0.98, *p* = 0.044). A similar effect estimate was observed for OS (HR = 0.35, 95% CI: 0.12–1.05), although statistical significance was not reached (*p* = 0.060).

## 4 Discussion

In this study, we systematically re‐evaluated the prognostic significance of MGMT promoter methylation in a large cohort of patients with IDH‐mutant gliomas within the framework of the WHO CNS 5 classification. By integrating quantitative methylation assessment, exploration of multiple cutoff thresholds, chemotherapy‐stratified subgroup analysis, and propensity score matching to address potential confounding, we provide a more nuanced interpretation of the role of MGMT in this molecularly defined subgroup.

Our findings demonstrate that MGMT promoter methylation is associated with improved survival in patients with IDH‐mutant gliomas. In the unmatched cohort, MGMT methylation emerged as an independent prognostic factor for both PFS and OS, consistent with several prior reports suggesting a favorable prognostic role in IDH‐mutant gliomas [5, 6, 7, 27]. After 1:1 propensity score matching, the direction and magnitude of these associations remained largely unchanged; however, statistical significance was no longer consistently observed. Notably, the hazard ratios remained comparable before and after matching, whereas the confidence intervals became relatively wider in the matched cohort. To further investigate whether the attenuation of statistical significance was primarily attributable to the reduced sample size, we performed additional PSM analyses using 1:2 and 1:3 matching ratios. Consistent with the results from 1:1 matching, no statistically significant survival differences were observed between MGMT‐methylated and MGMT‐unmethylated tumors in either the 1:2 or 1:3 matched cohorts. These findings suggest that the lack of statistical significance after PSM may not be solely attributable to the smaller matched sample size.

The importance of addressing confounding in retrospective glioma studies cannot be overstated. In our cohort, patients with MGMT methylation were more likely to harbor oligodendroglioma histology and present with more favorable preoperative performance status, all of which are well‐established determinants of improved outcome [28, 29, 30]. Without adequate adjustment, these imbalances may lead to overestimation of the prognostic value of MGMT methylation. By applying PSM, we were able to construct a more balanced comparison, thereby providing a more reliable estimate of its independent effect. This methodological approach represents a key strength of the present study and may partly explain discrepancies across prior reports.

Another important finding of our study is the absence of a clear quantitative association between the degree of MGMT methylation and survival outcomes. While patients with unmethylated tumors consistently exhibited worse prognosis, increasing methylation levels among methylated tumors did not translate into incremental survival benefits. This observation suggests that the biological and clinical relevance of MGMT methylation in IDH‐mutant gliomas may primarily reflect a binary “on–off” effect rather than a continuous dose–response relationship. Such findings have implications for the interpretation of pyrosequencing‐based assays and question the added value of fine‐grained quantitative stratification in this context.

In addition, we demonstrate that the prognostic performance of MGMT methylation is sensitive to the selection of cutoff values. While lower thresholds (e.g., 9%–15%) yielded significant associations with OS, higher cutoffs diminished this effect. This variability underscores the lack of standardized criteria for defining MGMT methylation positivity and highlights the need for harmonized analytical frameworks [10, 11, 31]. Importantly, our findings suggest that differences in cutoff selection across studies may contribute substantially to the inconsistent conclusions reported in the literature. Notably, differences observed between closely adjacent cutoff values should be interpreted with caution, as analytical variability inherent to pyrosequencing may introduce classification uncertainty for cases with methylation levels near predefined thresholds. Moreover, because MGMT methylation is inherently a continuous biological variable, dichotomization may result in loss of information and may not fully capture its relationship with clinical outcomes. Future studies incorporating continuous‐variable analyses and nonlinear modeling approaches may further refine the prognostic and predictive significance of quantitative MGMT methylation measurements.

As a predictive biomarker for alkylating chemotherapy, MGMT promoter methylation has been reported to primarily delay disease progression initially, with subsequent improvement in survival emerging over time [2]. In our stratified analyses, MGMT methylation was independently associated with prolonged OS exclusively among chemotherapy‐treated patients, consistent with previous clinical trial‐based reports [32, 33]. Although MGMT‐methylated patients demonstrated numerically longer PFS across all subgroups, these differences did not reach statistical significance, in line with prior observations [34]. The absence of significant PFS differences may be partly attributable to the difficulty in distinguishing pseudoprogression from true tumor progression based on retrospective imaging evaluations, as well as the impact of heterogeneous salvage treatments that may influence subsequent OS independently of initial treatment response. Collectively, these findings suggest that the predictive value of MGMT promoter methylation is primarily evident in patients receiving alkylating chemotherapy, whereas its prognostic significance in the absence of chemotherapy remains limited.

From a biological perspective, the context‐dependent prognostic significance of MGMT methylation in IDH‐mutant gliomas is biologically plausible. In contrast to IDH‐wildtype glioblastoma, IDH‐mutant tumors are characterized by a distinct epigenetic landscape, including the glioma CpG island methylator phenotype (G‐CIMP), which reflects widespread DNA hypermethylation and transcriptional reprogramming [35, 36]. Within this context, MGMT promoter methylation may represent only one component of a broader epigenetic network, thereby limiting its relative contribution as an independent determinant of clinical outcome [37]. Furthermore, DNA repair capacity in IDH‐mutant gliomas is regulated by multiple pathways beyond MGMT. Alkylation‐induced DNA lesions can be processed through base excision repair and mismatch repair systems, both of which influence cellular responses to temozolomide, resulting in the prolonged survival of the chemotherapy‐treated patients [38, 39]. Alterations in mismatch repair, particularly involving MSH6, have been associated with treatment resistance and hypermutation in gliomas [2, 40]. Moreover, the globally altered epigenetic state induced by IDH mutations may further modulate DNA damage response pathways [41, 42]. Taken together, these factors suggest that MGMT methylation is unlikely to function as a dominant, standalone biomarker in this molecular subgroup, but rather as a context‐dependent modifier whose impact is shaped by interactions with other biological and clinical variables.

Several limitations of this study should be acknowledged. First, owing to the limited sample size (*n* = 11), the Oligo−CHT subgroup was not included in survival analyses, representing an important limitation that precludes definitive conclusions regarding the prognostic significance of MGMT in untreated oligodendroglioma patients. Second, its retrospective design inherently introduces potential selection bias, despite the use of PSM. Third, treatment heterogeneity across patients may influence outcomes and cannot be fully controlled. Fourth, the relatively limited follow‐up duration means that a proportion of patients have not yet reached the study endpoints; therefore, the conclusions drawn from the current analysis require further validation. Ongoing follow‐up will be conducted to reassess and strengthen the robustness of our findings. Finally, external validation in independent cohorts is warranted to confirm the generalizability of our findings.

In conclusion, MGMT promoter methylation is associated with favorable clinical outcomes in patients with IDH‐mutant gliomas; however, its prognostic significance appears to be influenced by treatment context, molecular subtype, and methodological factors such as cutoff selection. The direction and magnitude of the association remained generally consistent after adjustment for baseline imbalances, although statistical significance was less stable in the matched cohort. These findings support a context‐dependent interpretation of MGMT methylation and highlight the need for standardized analytical strategies and integrated biomarker assessment in diffuse gliomas.

## Author Contributions

Acquisition of data: H.J., L.C., X.C., S.T., Q.Z., J.Y., and S.L.; Analysis and Interpretation of Data: H.J., L.C., X.C., S.T., and Q.Z.; Statistical analysis: H.J., L.C., X.C., and Y.C.; Drafting the Article: H.J., L.C., and X.C.; Critically Revising the Article: H.J., L.C., and Y.C.; funding acquisition: H.J.; Conception and Design: H.J., S.L., and Y.C.; and study supervision: H.J. and Y.C.

## Funding

This work was supported by the National Natural Science Foundation of China (82202983), the Beijing Natural Science Foundation (L252079), and the Peking University Clinical Scientist Training Program, supported by the Fundamental Research Funds for the Central Universities (BMU2024PYJH002).

## Ethics Statement

This study conformed to the ethical guidelines of the 1975 Declaration of Helsinki and was approved by the Institutional Review Board of BTH.

## Consent

The authors have nothing to report.

## Conflicts of Interest

The authors declare no conflicts of interest.

## Supporting information


**Figure S1:** Molecular classification of diffuse gliomas and study inclusion criteria.
**Figure S2:** Kaplan–Meier survival curves stratified by MGMT methylation after propensity score matching.
**Table S1:** Cox regression analysis using MGMT methylation percentage as a continuous variable.
**Table S2:** Comparison of clinical data between methylated and unmethylated gliomas after 1:2 PSM.
**Table S3:** Comparison of clinical data between methylated and unmethylated gliomas after 1:3 PSM.

## Data Availability

The data that support the findings of this study are available from the corresponding author upon reasonable request.
