# Supplementary material for: Clinical Impact of MGMT Promoter Methylation in IDH‐Mutant Gliomas: Influence of Threshold Selection and Clinical Confounding
Source: Ann Clin Transl Neurol. 2026 Sep 8:10.1002/acn3.70528. Online ahead of print. doi: 10.1002/acn3.70528 (PMC13554488; doi:10.1002/acn3.70528)
Supplement: Supplementary file 1 [file ACN3-9999-0-s001.zip › acn370528-sup-0002-FigureS2.pdf]

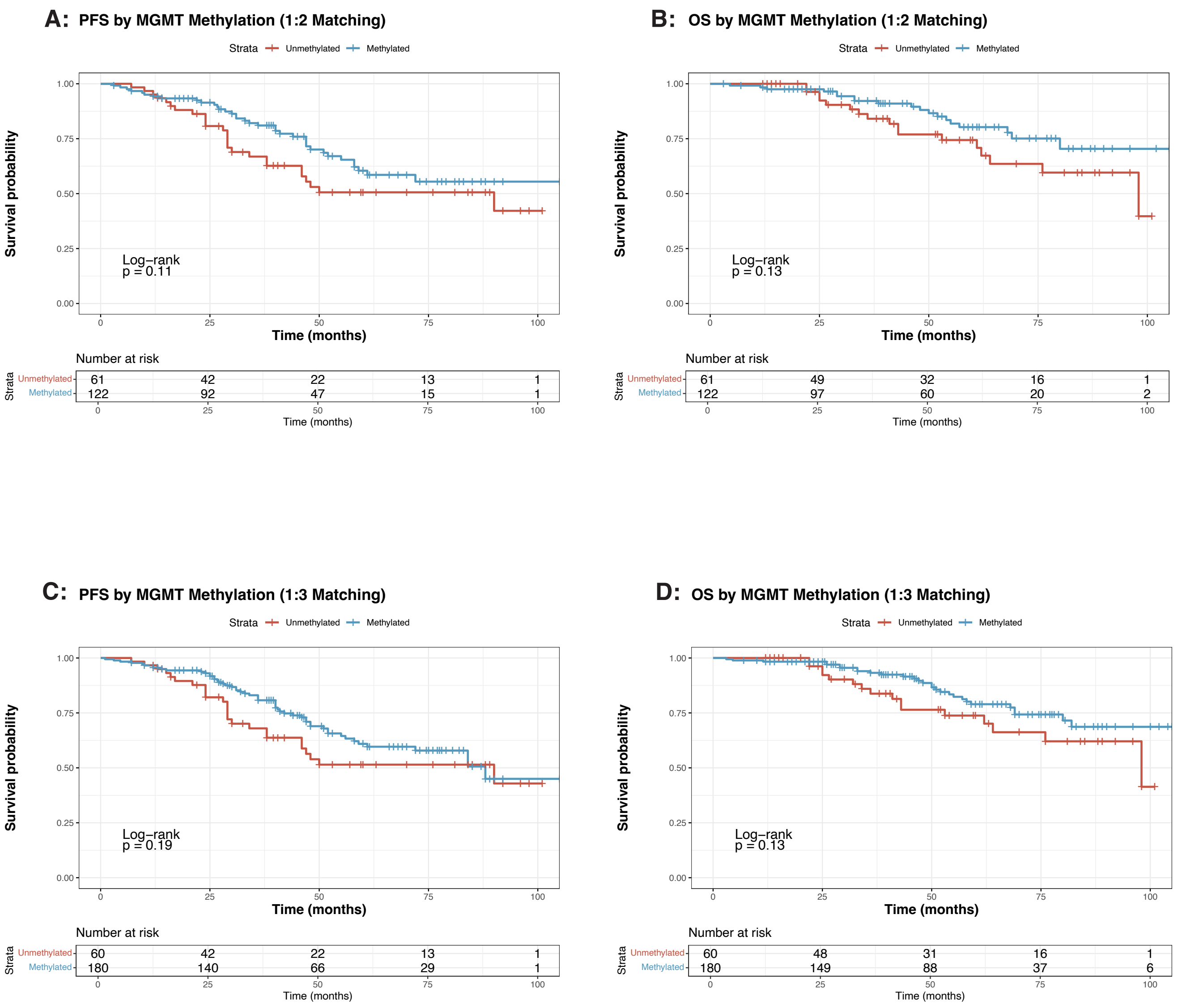
A: PFS by MGMT Methylation (1:2 Matching)
Strata
Unmethylated
Methylated
Survival probability
1.00
0.75
0.50
0.25
0.00
Log−rank
p = 0.11
0
25
50
75
100
Time (months)
Number at risk
Strata
Unmethylated 61 42 22 13 1
Methylated 122 92 47 15 1
Time (months)
B: OS by MGMT Methylation (1:2 Matching)
Strata
Unmethylated
Methylated
Survival probability
Log−rank
p = 0.13
Time (months)
Number at risk
Strata
Unmethylated 61 49 32 16 1
Methylated 122 97 60 20 2
Time (months)
C: PFS by MGMT Methylation (1:3 Matching)
Strata
Unmethylated
Methylated
Survival probability
Log−rank
p = 0.19
Time (months)
Number at risk
Strata
Unmethylated 60 42 22 13 1
Methylated 180 140 66 29 1
Time (months)
D: OS by MGMT Methylation (1:3 Matching)
Strata
Unmethylated
Methylated
Survival probability
Log−rank
p = 0.13
Time (months)
Number at risk
Strata
Unmethylated 60 48 31 16 1
Methylated 180 149 88 37 6
Time (months)
