# Supplementary material for: Clinical Impact of MGMT Promoter Methylation in IDH‐Mutant Gliomas: Influence of Threshold Selection and Clinical Confounding
Source: Ann Clin Transl Neurol. 2026 Sep 8:10.1002/acn3.70528. Online ahead of print. doi: 10.1002/acn3.70528 (PMC13554488; doi:10.1002/acn3.70528)
Supplement: Supplementary file 1 [file ACN3-9999-0-s001.zip › acn370528-sup-0003-TableS1.docx]

TABLE S1 Cox regression analysis using MGMT methylation percentage as a continuous variable

| MGMT | Hazard ratio | 95% confidence interval | *P* value |
| --- | --- | --- | --- |
| Before PSM |  |  |  |
| In terms of PFS | 0.984 | 0.971-0.997 | 0.014 |
| In terms of OS | 0.995 | 0.978-1.012 | 0.578 |
| After PSM |  |  |  |
| In terms of PFS | 0.977 | 0.952-1.003 | 0.082 |
| In terms of OS | 0.987 | 0.956-1.019 | 0.419 |
