# Supplementary material for: Clinical Impact of MGMT Promoter Methylation in IDH‐Mutant Gliomas: Influence of Threshold Selection and Clinical Confounding
Source: Ann Clin Transl Neurol. 2026 Sep 8:10.1002/acn3.70528. Online ahead of print. doi: 10.1002/acn3.70528 (PMC13554488; doi:10.1002/acn3.70528)
Supplement: Supplementary file 1 [file ACN3-9999-0-s001.zip › acn370528-sup-0004-TableS2.docx]

TABLE S2 Comparison of clinical data between methylated and unmethylated gliomas after 1:2 PSM

| Variable | Unmethylated  (n=61) | Methylated  (n=122) | *P* value |
| --- | --- | --- | --- |
| Age (year) | 39.3±11.8 | 39.8±8.8 | 0.788 |
| Gender (n, %) |  |  | 0.530 |
| Male | 34 (55.7%) | 62 (50.8%) |  |
| Female | 27 (44.3%) | 60 (49.2%) |  |
| KPS score | 81.0±11.1 | 80.1±12.9 | 0.641 |
| Location (n, %) |  |  | 0.245 |
| Left hemisphere | 21 (34.4%) | 54 (44.3%) |  |
| Right hemisphere | 34 (55.7%) | 52 (42.6%) |  |
| Bilateral | 6 (9.8%) | 16 (13.1%) |  |
| Tumor size (n, %) |  |  | 0.599 |
| 1 lobe | 31 (50.8%) | 71 (58.2%) |  |
| 2 lobes | 18 (29.5%) | 32 (26.2%) |  |
| 3 lobes | 10 (16.4%) | 13 (10.7%) |  |
| ≥4 lobes | 2 (3.3%) | 6 (4.9%) |  |
| Pathology (n, %) |  |  | 0.802 |
| 1p/19q co-deleted and IDH-mut oligodendrogliomas | 13 (21.3%) | 28 (23.0%) |  |
| IDH-mut astrocytoma | 48 (78.7%) | 94 (77.0%) |  |
| Tumor grade (n, %) |  |  | 0.738 |
| WHO 2 | 40 (65.6%) | 83 (68.0%) |  |
| WHO 3 | 21 (34.4%) | 39 (32.0%) |  |
| Extent of resection (n, %) |  |  | 0.850 |
| Supratotal | 3 (4.9%) | 7 (5.7%) |  |
| Total | 21 (34.4%) | 45 (36.9%) |  |
| Subtotal | 25 (41.0%) | 42 (34.4%) |  |
| Partial | 12 (19.7%) | 28 (23.0%) |  |
| Chemotherapy (n, %) |  |  |  |
| Yes | 46 (75.4%) | 75 (61.5%) | 0.072 |
| No | 15 (24.6%) | 41 (33.6%) |  |
| NA | 0 (0.0%) | 6 (4.9%) |  |
| Radiotherapy (n, %) |  |  | 0.061 |
| Yes | 41 (67.2%) | 65 (53.3%) |  |
| No | 20 (32.8%) | 50 (41.0%) |  |
| NA | 0 (0.0%) | 7 (5.7%) |  |

Abbreviations: PSM, propensity score matching; KPS, karnofsky performance score; IDH, isocitrate dehydrogenase; WHO, World Health Organization; NA, not available.
