# Supplementary material for: Clinical Impact of MGMT Promoter Methylation in IDH‐Mutant Gliomas: Influence of Threshold Selection and Clinical Confounding
Source: Ann Clin Transl Neurol. 2026 Sep 8:10.1002/acn3.70528. Online ahead of print. doi: 10.1002/acn3.70528 (PMC13554488; doi:10.1002/acn3.70528)
Supplement: Supplementary file 1 [file ACN3-9999-0-s001.zip › acn370528-sup-0005-TableS3.docx]

TABLE S3 Comparison of clinical data between methylated and unmethylated gliomas after 1:3 PSM

| Variable | Unmethylated  (n=60) | Methylated  (n=180) | *P* value |
| --- | --- | --- | --- |
| Age (year) | 39.2±11.8 | 39.7±9.5 | 0.704 |
| Gender (n, %) |  |  | 0.881 |
| Male | 33 (55.0%) | 101 (56.1%) |  |
| Female | 27 (45.0%) | 79 (43.9%) |  |
| KPS score | 81.2±11.1 | 81.3±12.6 | 0.952 |
| Location (n, %) |  |  | 0.105 |
| Left hemisphere | 21 (35.0%) | 82 (45.6%) |  |
| Right hemisphere | 33 (55.0%) | 71 (39.4%) |  |
| Bilateral | 6 (10.0%) | 27 (15.0%) |  |
| Tumor size (n, %) |  |  | 0.696 |
| 1 lobe | 30 (50.0%) | 96 (53.3%) |  |
| 2 lobes | 18 (30.0%) | 47 (26.1%) |  |
| 3 lobes | 10 (16.7%) | 25 (13.9%) |  |
| ≥4 lobes | 2 (3.3%) | 12 (6.7%) |  |
| Pathology (n, %) |  |  | 1.0 |
| 1p/19q co-deleted and IDH-mut oligodendrogliomas | 13 (21.7%) | 39 (21.7%) |  |
| IDH-mut astrocytoma | 47 (78.3%) | 141 (78.3%) |  |
| Tumor grade (n, %) |  |  | 1.0 |
| WHO 2 | 40 (66.7%) | 120 (66.7%) |  |
| WHO 3 | 20 (33.3%) | 60 (33.3%) |  |
| Extent of resection (n, %) |  |  | 0.812 |
| Supratotal | 3 (5.0%) | 8 (4.4%) |  |
| Total | 21 (35.0%) | 67 (37.2%) |  |
| Subtotal | 25 (41.7%) | 64 (35.6%) |  |
| Partial | 11 (18.3%) | 41 (22.8%) |  |
| Chemotherapy (n, %) |  |  |  |
| Yes | 45 (75.0%) | 118 (65.6%) | 0.161 |
| No | 15 (25.0%) | 54 (30.0%) |  |
| NA | 0 (0.0%) | 8 (4.4%) |  |
| Radiotherapy (n, %) |  |  | 0.101 |
| Yes | 40 (66.7%) | 99 (55.0%) |  |
| No | 20 (33.3%) | 72 (40.0%) |  |
| NA | 0 (0.0%) | 9 (5.0%) |  |

Abbreviations: PSM, propensity score matching; KPS, karnofsky performance score; IDH, isocitrate dehydrogenase; WHO, World Health Organization; NA, not available.
